# Mapping the Knowledge of Antipsychotics-Induced Sudden Cardiac Death: A Scientometric Analysis in CiteSpace and VOSviewer

**DOI:** 10.3389/fpsyt.2022.925583

**Published:** 2022-07-07

**Authors:** Min Wang, Yixun Ma, Zefang Shen, Lufang Jiang, Xiaoyuan Zhang, Xuan Wei, Zhengqi Han, Hongxia Liu, Tiantong Yang

**Affiliations:** ^1^Key Laboratory of Evidence Science, Institute of Evidence Law and Forensic Science, Ministry of Education, China University of Political Science and Law, Beijing, China; ^2^Collaborative Innovation Center of Judicial Civilization, Beijing, China; ^3^Institute for Digital Technology and Law, China University of Political Science and Law, Beijing, China; ^4^The CUPL Scientometrics and Evaluation Center of Rule of Law, China University of Political Science and Law, Beijing, China

**Keywords:** sudden cardiac death, antipsychotic, scientometric analysis, CiteSpace, VOSviewer

## Abstract

The drugs on the market for schizophrenia are first-generation and second-generation antipsychotics. Some of the first-generation drugs have more side effects than the other drugs, so they are gradually no longer being applied clinically. Years of research have shown that the risk of sudden cardiac death in psychotic patients is associated with drug use, and antipsychotic drugs have certain cardiotoxicity and can induce arrhythmias. The mechanism of antipsychotic-induced sudden cardiac death is complicated. Highly cited papers are among the most commonly used indicators for measuring scientific excellence. This article presents a high-level analysis of highly cited papers using Web of Science core collection databases, scientometrics methods, and thematic clusters. Temporal dynamics of focus topics are identified using a collaborative network (author, institution, thematic clusters, and temporal dynamics of focus topics are identified), keyword co-occurrence analysis, co-citation clustering, and keyword evolution. The primary purpose of this study is to discuss the visual results, summarize the research progress, and predict the future research trends by bibliometric methods of CiteSpace and VOSviewer. This study showed that a research hotspot is that the mechanisms of cardiotoxicity, the safety monitoring, and the assessment of the risk-benefit during clinical use of some newer antipsychotics, clozapine and olanzapine. We discussed relevant key articles briefly and provided ideas for future research directions for more researchers to conduct related research.

## Introduction

Sudden death is one of the leading causes of death in young people and is also not uncommon in older people and children (1, 2). Sudden death can be divided into cardiac and non-cardiac sudden death, with the former accounting for 70% of sudden death cases. Therefore, the study of sudden cardiac death is a major focus of research in both clinical and forensic medicine. Studies on the causes and risk factors of sudden cardiac death have been more comprehensive, including studies on the genetic predispositions, sex, age, own diseases (such as coronary heart disease), and lifestyle habits of the deceased (3). Some studies have shown that antipsychotic medications have a wide range of adverse effects, such as mild sedation or dry mouth, constipation, inability to sit still, sexual dysfunction, acute dystonia, weight gain, delayed dyskinesia, myocarditis, and granulocyte deficiency (4). The degree of response ranges from relatively mild and imperceptible tolerance problems to clear unpleasant sensations, and from mild somatic pain to disfigurement and even life-threatening symptoms. Additionally, numerous related studies have shown that drug use can directly increase the risk of sudden cardiac death (5). Researchers in 2020 noted that increased mortality was observed in patients with mental health disorders. Antipsychotic medications can carry a greater than two-fold risk of sudden death, which is thought to be mediated through effects on QT interval prolongation and risk of tip-twist ventricular tachycardia (6). Antipsychotics have been popular in clinical use for decades, but considerable difficulties and challenges remain in reducing the risk associated with sudden cardiac death. Among second-generation antipsychotics, clozapine and olanzapine are the two most prescribed in clinic practice, which have been proved to be related to cardiotoxicity. The dual-omics study integrating proteome and transcriptome analyses identified the spliceosome signaling as the common mechanism behind clozapine and other TGAs that induce cardiotoxicity (7). A study based on zebrafish embryos has shown that, clozapine can increase the levels of reactive oxygen species and a lipid peroxidation marker leading to oxidative and inflammation, thus resulting in cardiotoxicity (8). Another study based on male rats showed that, in the rat heart, olanzapine can increase the level of acetyl-CoA carboxylase phosphorylation and tissue ATP level, also reduce the level of Akt and its downstream products AS160, which may be involved with the mechanism for the adverse cardiac effects (9). Data from a Italian study showed that the elderly frail subjects were more likely to be prescribed second-generation antipsychotics and this may be involved in cardiometabolic disorders and other comorbidities (10). The antipsychotics are often used off-label among the patients with Alzheimer’s disease in terms of indications for use, dose and route of administration (11), this may increase the risk of sudden cardiac death induced by antipsychotics. Recently, newer antipsychotic drugs have been introduced into the clinic practice, such as brexpiprazole, cariprazine, and lumateperone, which have a higher safety performance and fewer metabolic effects than second-generation antipsychotics. The emerging efficacy and tolerability data were also a promising research direction (12). It was also reported that the third-generation antipsychotics (TGAs) with favorable metabolic outcomes could have large potential of clinical practice (13). Therefore, the clinical use of antipsychotics is evaluated for risk and benefit, and their risk effects are considered first and foremost.

In the era of big data, information is complex and fragmented. Many studies related to sudden cardiac death caused by antipsychotic drugs and the effects of these drugs on the heart have been conducted over time. A more systematic and comprehensive review of studies may be necessary. Using bibliometric methods to visually analyze the collated literature, the traditional method of reading a large amount of literature can be eliminated, omissions and subjective bias can be reduced, more time can be saved, and more accurate results can be obtained. This article examines the use of visual analytics to mine and analyze the important research literature, and the new content can provide some convenience for subsequent researchers.

## Materials and Methods

### Data Collection

Web of Science (WOS) is one of the largest and most prestigious databases in the world. Currently, the WOS database has been widely used for bibliometric research. In this study, the WOS core database was used as the research data source. “Antipsychotic” and “Sudden Cardiac Death” were used as the core subject terms, and the search formula was grouped after expanding the related vocabulary without selecting the time span, refining the literature types as original research articles and review articles, and cleaning the literature categories to obtain 999 documents. All the information associated with the abovementioned literature was exported in text format to generate a dataset for this study. The search formula based on this study grouping was as follows:

TS = (Antipsychotic OR Chlorpromazine OR Droperidol OR Inapsine OR Fluphenazine OR Haloperidol OR Haldol OR Loxapine OR Perphenazine OR Pimozide OR ORAP OR Prochlorperazine OR Thioridazine OR Mellaril OR Thiothixene OR Navane OR Trifluoperazine OR Aripiprazole OR Abilify OR Asenapine OR Saphris OR Clozapine OR Clozaril OR Iloperidone OR Fanapt OR Olanzapine OR Zyprexa OR Lurasidone OR Latuda OR Paliperidone OR Invega OR Quetiapine OR Seroquel OR Risperidone OR “Risperdal consta” OR Ziprasidone OR Geodon) AND TS = (“sudden cardiac death” OR myocarditis OR “acute myocardial infarction” OR arrhythmia OR “ventricular tachycardia” OR “sinus bradycardia” OR “conduction disorder” OR “heart failure” OR cardiomyopathy OR “ventricular fibrillation and flutter” OR “atrioventricular block”).

It should be noted that the above searchable terms are derived from the Tables of FDA-Approved Indications for First- and Second-Generation Antipsychotics and the International Classification of Diseases provided by the United States National Library of Medicine.

### Data Analysis

#### Collaborative Network Analysis

Collaborative network analysis is carried out based on social network analysis theory, which originated from anthropological and sociological exploration of interpersonal relationships in complex social clusters (14). If several countries, institutions, and authors have published collaborative literature together, it can be concluded that there is a collaborative relationship between them, and multiple collaborative relationships can form a larger collaborative network in the field. Such a collaborative network analysis can help researchers understand the current overall social factors in the field.

#### Co-occurrence Analysis

From the frequencies of subject terms and keywords in a group of literature, a co-occurrence map of subject terms and keywords in the field can be drawn, reflecting the macroscopic research status and relationships among disciplines and topics in the field.

#### Cluster Analysis

Clustering analysis applies the idea of dimensionality reduction to group a collection of physical or abstract objects into multiple classes consisting of comparable objects to describe similarities between different data sources.

#### Citation Analysis

After analyzing the clustering results, we delve into the citations given and cited in the important clusters. By understanding the research base and frontier, we can sort out the research direction, dynamic evolution process, and existence of patterns.

#### Keyword Evolution

The frequency of keyword clustering analysis on the timeline can indicate the research hotspots. The combination of keywords and time can learn which keywords appeared in the same year horizontally and lock in multiple perspectives of research in that year. New, retained, and lost keywords vertically can show the research development pulse and help predict the future research direction.

## Results

### General Information

**FIGURE 1:**
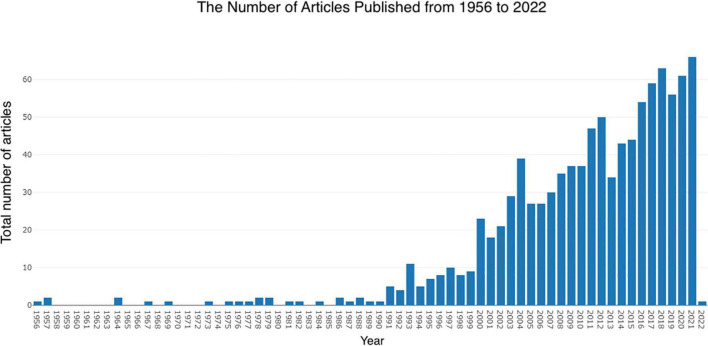
Development of the number of articles over the years.

**FIGURE 2:**
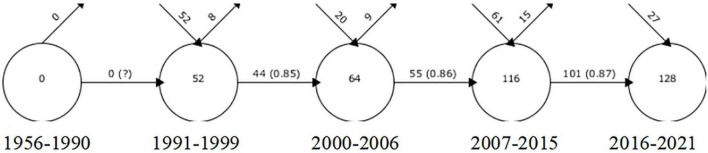
Overlapping map.

In total, 999 papers were retrieved using the search formula in the WOS database after filtering by requirement. The timespan was from 1956 to 2022. The year 2021 had the highest number of publications, followed by 2018, with 66 and 63, respectively (Figure 1). Overall, 52 key terms were added to the previous year interval between 1991 and 1999. Eight terms were lost to the next year interval between 2000 and 2006, 44 terms were retained, while 20 new terms were added during the 8 years from 2007 to 2015. The number of words retained in the previous year interval was 55, with the number of fresh words increasing by 61 and 15 words lost. Of these, 101 words were retained until the interval 2016 to 2021, and together with the 27 new words, a total of 128 words with strong thematic connections were retained to date (Figure 2).

**FIGURE 3:**
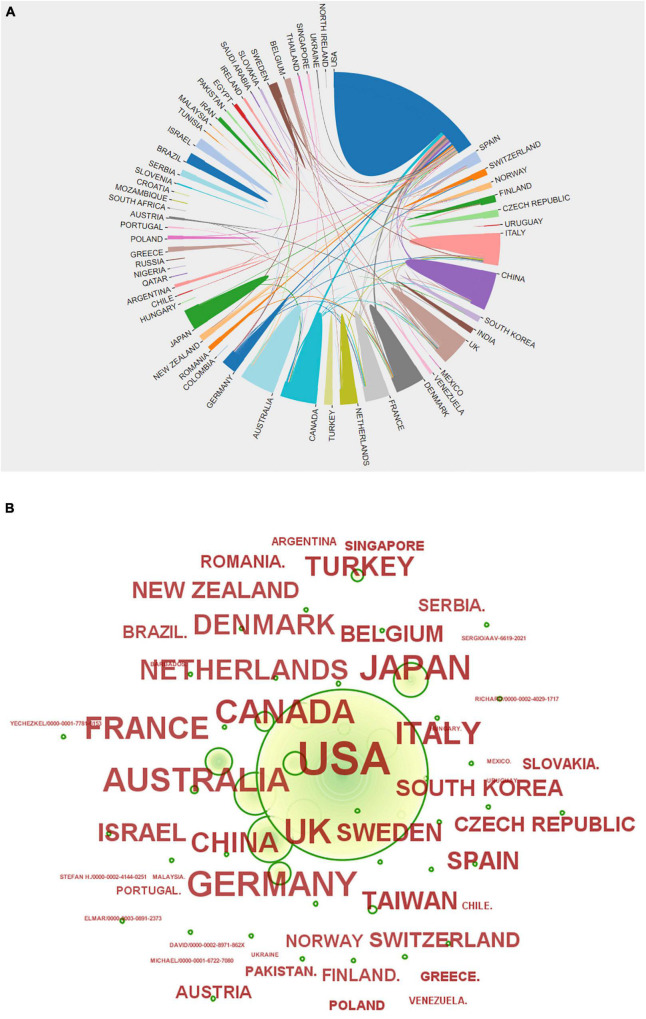
**(A)** Country network visualization showing connections among countries. **(B)** Various countries’ issuance status.

The United States of America, United Kingdom, and Germany ranked as the top three in the distribution of the total number of articles issued, followed by Japan, Australia, Canada, Italy, and France. China ranked ninth, but the number of articles published is similar to that of the top countries. Additionally, the overall number is increasing year by year, with a tendency to be among the top research countries in the world. For details, see Figure 3 and Table 1.

**TABLE 1:** Number of articles published in the top 12 countries.

Rank	Counts	Countries	Mean (Year)
1	265	UNITED STATES	1976
2	60	UNITED KINGDOM	1973
3	58	GERMANY	1994
4	55	JAPAN	1981
5	50	AUSTRALIA	1995
6	47	CANADA	1993
7	39	ITALY	1995
8	38	FRANCE	1993
9	31	CHINA	2007
10	31	NETHERLANDS	2001
11	30	DENMARK	2002
12	21	TURKEY	2006

*Mean (Year), the mean of the years when the literature published.*

Figure 3 shows that there are extensive collaborations among various countries in studies related to sudden cardiac death from antipsychotics. Of these, the United States has the most collaborations and close ties with other countries. China has the most ties with the United States, followed by those with the United Kingdom and Canada, and then with other countries. However, the top countries in the world in terms of number of publications, such as the United Kingdom, Germany, Canada, Italy, and France, have extensive ties with other countries. Although there is no direct positive correlation between the number of publications and national partnerships, it seems that effective and strong ties will promote research development as shown by the partnerships among the top countries in terms of the number of publications. Therefore, China can strengthen more collaborative exchanges with other countries.

### Research Clusters

**FIGURE 4:**
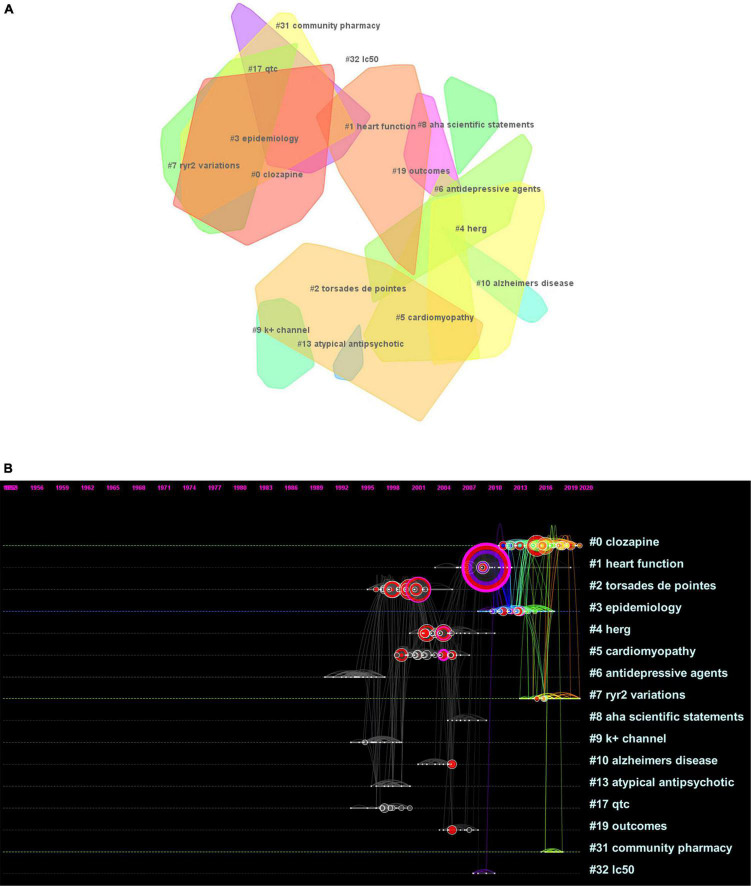
**(A)** Landscape view of the co-citation network. All cluster labels were extracted from titles of citing articles using the log-likelihood ratio algorithm. **(B)** Timelines of co-citation clusters. Major clusters are labeled on the right.

In total, 16 clusters were generated in the literature, as detailed in Figure 4A and Table 2. According to the average time of cluster formation, clusters 0, 3, 7, and 31 were formed relatively close to each other, and cluster 6 was formed earliest. To some extent, this indicates that researchers have started to focus on some effects of antipsychotic drugs on somatic toxicity as early as 1989 or even earlier. Recent advances have refined the disease classification and pathogenesis, and focused on the side effects of drugs.

**TABLE 2:** Statistics of different clusters in Figure 4A.

Cluster	Counts	Silhouette	Mean (Year)	Label
0	104	0.95	2016	Clozapine
1	96	0.969	2009	Heart function
2	91	0.918	1999	Torsade de Pointes
3	80	0.917	2012	Epidemiology
4	66	0.918	2004	hERG
5	60	0.859	2002	Cardiomyopathy
6	35	0.955	1993	Antidepressant agents
7	33	0.965	2016	RYR2 variations
8	30	0.994	2006	aha scientific statements
9	25	0.946	1996	K + channel
10	24	0.973	2003	Alzheimer’s disease
13	19	0.997	1998	Atypical antipsychotic
17	15	0.98	1997	QTc
19	14	0.993	2006	Outcomes
31	6	0.997	2016	Community pharmacy
32	6	0.999	2008	IC50

*Silhouette (S-value), an evaluation of clustering validity, is based on the comparison of its tightness and separation. When S > 0.5, it means that the clustering is justified; when S > 0.7, it means that the clustering is convincing.*

The timeline plot (Figure 4B) shows that clusters 0 and 7 have long durations and good continuity. Clusters 2 and 4 have fewer recent publications but have long durations, followed by cluster 5. The remaining clusters have no articles published for a longer period of time, where clusters 8, 10, 13, 19, and 32 have short durations, cluster 32 has a weak association with other clusters, and cluster 10 is cited with high frequency. The top ranked item by citation counts is Ray et al. (15) in cluster 6, with 59 citations. The second and third are Reilly (16) and Glassman (17), respectively, in cluster 0, with 29 citations. The 4th is Ronaldson et al. (18) in Cluster #3, with citation counts of 22. The fifth is Curto et al. (19) in cluster 3, with 19 citations. The sixth is Ray (20) in cluster 0, with 18 citations. The 7th is Haddad (21) in Cluster #0, with citation counts of 17. The eighth is Drici (22) in cluster 0, with 17 citations. The ninth is Welch (23) in cluster 0, with 15 citations. The tenth is Roden et al. (24) in cluster 8, with 15 citations.

From the above results, we selected the most closely related citing articles and cited references from the three clusters (clusters 0, 1, and 7) needed for this study, and further described and analyzed the citations within each cluster.

#### Cluster 0 “Clozapine”

Cluster 0 has the largest number of clusters in studies related to cardiac effects, such as sudden cardiac death from antipsychotic drugs. The main focus of the study is the cardiotoxicity of the drug clozapine, and this adverse effect is non-specific. This cluster, however, is based on clinical trials conducted in patients using the antipsychotic drug clozapine, with electrocardiographic observations of clozapine-induced myocarditis and cardiomyopathy as an assessment of the risk-benefit of drug use. Among the literature most closely related to this cluster, Ronaldson et al. (18), Curto et al. (19), Bellissima et al. (25), Knoph et al. (26), and Ronaldson et al. (27) have the highest citation frequency and can represent the research base in this field. The top five cited studies, Barnes et al. (28), Chopra et al. (29), Kanniah et al. (30), Brazile et al. (31), and Anıl Yağcıoğlu et al. (32), can represent the research frontiers in this field (Table 3).

**TABLE 3:** Cited references and citing articles of Cluster #0 clozapine.

Cluster #0 clozapine		
**Cited references**	**Citing articles**	
**References**	**Coverage %**	**References**
(18)	15	(28)
(19)	10	(29)
(25)	9	(30)
(26)	8	(31)
(27)	8	(32)

*Coverage, the proportion of the cited literature of this Citing Article in the cluster. The larger the value is, the higher the relevance of the article to the topic clusters, indicating that more topics can be found in that article.*

#### Cluster 1 “Heart Function”

Cluster 1 studies have focused on the effects of antipsychotics on cardiac function. Among the literature most closely related to this cluster, Ray et al. (15), Tiihonen et al. (33), Haas et al. (34), Leucht et al. (35), and Nuttall et al. (36) have the highest frequency of citations and can represent the research base in this field. The top five cited studies, Poluzzi et al. (37), Poluzzi et al. (37), Gunnstroem et al. (38), Mehta et al. (39), and Ababneh et al. (40), can represent the research frontiers in the field (Table 4).

**TABLE 4:** Cited references and citing articles of Cluster #1 heart function.

Cluster #1 heart function		
**Cited references**	**Citing articles**	
**References**	**Coverage %**	**References**
(15)	16	(37)
(33)	11	(37)
(34)	10	(38)
(35)	8	(39)
(36)	8	(40)

#### Cluster 7 “Drug-Induced QT Interval Prolongation”

Cluster 7 was formed the latest on average, first appearing in 2013 and lasting for 8 years, indicating that the cluster is innovative compared with previous studies. The main focus of this cluster study was drug-induced QT interval prolongation. The studies include ECG characteristics and clinical treatment. Among the literature most closely related to this cluster, Wu et al. (41), Vandenberk et al. (42), Salvo et al. (43), Roden et al. (44), and Schwartz and Woosley, (45) have the highest citation frequency and can represent the research base in this field. The top five Schwartz citations, Aroke et al. (46), Xiong et al. (47), Pelletti et al. (48), Aronow and Shamliyan, (49), and Christiansen et al. (50), can represent the research frontiers in the field (Table 5).

**TABLE 5:** Cited references and citing articles of Cluster #7 RYR2 variations.

Cluster #7 RYR2 variations		
**Cited references**	**Citing articles**	
**References**	**Coverage %**	**References**
(41)	6	(46)
(42)	5	(47)
(43)	5	(48)
(44)	5	(49)
(45)	4	(50)

### Keyword Evolution

Keywords can systematically and intuitively present the focus and “hotness” of a certain type of research topic. By observing the evolution of keywords in the time dimension, we can dig deeper into the research content of important literature, such as the pulse of that research direction. The position of the keyword is the year of its first appearance. As the subsequent interest rises, the frequency of the keyword increases, thus presenting a larger circle in the graph. The timeline graph also shows the evolution and development of keywords under each cluster, thus summarizing the current status of research development and predicting the next research trends.

Typically, research themes go through three stages: incubation, development, and maturation. The number of articles over the years can visually show the historical results and the evolution of keywords under the topic of the cluster, and can also be more helpful in illustrating how the information within the cluster is interconnected and influential. It is worthwhile to explore the following information: the year when the first article appeared, the year when the literature started to increase, the year when the attention and interest started to decrease and cool down, the year when the landmark literature appeared, and the year when the highly cited and highly mediated centralized literature appeared. The goal is to understand how the literature influenced the whole trend of the cluster.

**FIGURE 5:**
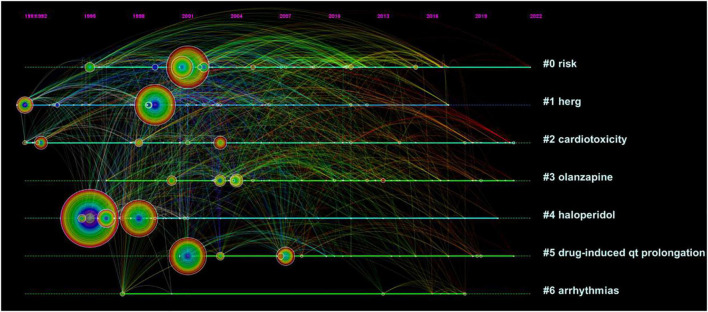
Keyword timeline view.

To clearly present the keyword evolution characteristics of the topic of this study, the time interval was chosen to be 2 years and the g-index algorithm was selected, the parameter *k* = 20 was set, the threshold was adjusted downward, and then post-run clustering was run. Then, the clustering labels were named with keywords and a timeline graph was generated after important keyword merging was performed. The system generates a total of nine clusters and chooses to present the first seven clusters that are larger, more connected, and have higher continuity. The themes of the seven clusters from clusters 0 to 6 were: risk, hERG, cardiotoxicity, clozapine, haloperidol, drug-induced QT prolongation, and arrhythmias. Combined with the number of articles published over the years, studies on the effects of antipsychotics on the heart or causing sudden cardiac death began in 1956 or even earlier. Before 1991, the volume of literature did not increase much and the total number of articles was only around 10. Therefore, no keywords could be extracted. From 1991 to 2021, the vast majority of keywords with higher word frequency were concentrated before 2008, likely indicating that researchers have been relatively mature in their research on the effects of antipsychotics on the heart or even causing sudden cardiac death. Based on this, clusters 0, 1, and 5, in which the keywords with high values of mediated centrality were located, were selected for analysis (Figure 5).

Cluster #0 theme is “risk” (Supplementary Figure 1A). From left to right, the keywords are: antidepressant drug, cardiotoxicity, death, diagnosis, acute myocardial infarction, sudden death, disorder, abnormality, mortality, QT interval, clozapine, atypical antipsychotics, interval abnormality, sex, antidepressant, accuracy, dementia, psychotropic drug, bipolar, trial, ventricular arrhythmia, cardiac arrest, schizophrenia patient, toxicity, elderly patient, positron emission, tomography, serotonin reuptake inhibitor, medication, care, drug use, older adult, cerebrovascular event, admission, acute confusional states, nursing home resident, London, agranulocytosis, meta-analysis, QTc interval prolongation, pneumonia, follow up, delirium, and intensive care unit.

Clustering theme #1 is “hERG” (Supplementary Figure 1B). From left to right, the keywords are: cell, anesthesia, arrhythmia, quinidine, channel, cardiac arrhythmia, calcium channel, activated potassium conductance, cancer, chemotherapy, chlorpromazine, agent, dofetilide, terfenadine, block, hERG, long QT syndrome, prolongation, potassium channel, QT interval, atrioventricular junction, action potential duration, repolarization, age, iKr, *in vivo*, rectifier, cisapride, dispersion, K + channel, metabolite, current, amisulpride, antiarrhythmic agent, anterior pituitary, QTc, rat, modulation, calcium, heart rate, cardiac repolarization, repolarization reserve, rabbit, pharmacology, antagonism, and clinical trial.

The theme of cluster #5 is “drug-induced QT prolongation” (Supplementary Figure 1C). From left to right, the keywords are: ventricular tachycardia, antipsychotic drug, risk factor, brain natriuretic peptide, prevalence, sudden cardiac death, adult, menon, antianginal agent, atrial fibrillation, non-steroidal anti-inflammatory drug, prescription, first trimester pregnancy, first trimester, psychiatric patient, all-cause mortality, antipsychotic medication, pattern, constipation, epidemiology, knowledge, long QT, and age.

### The R Programming Language-Thematic Map

R language topic maps can be combined with research topics to categorize and select the topics we need to engage with while predicting related research trends. The topic map is divided into four quadrants, which are clear and intuitive with distinct meanings. The first quadrant indicates themes that are both important and well developed; the second quadrant indicates themes that are well developed but not important to the current field; the third quadrant is for marginal themes that are not well developed, may have just emerged, and may be disappearing; and the fourth quadrant indicates themes that are important to the field but not well developed. The data suggest that research related to QT interval prolongation syndrome, KCNJ gene, arrhythmia, tip-twisting ventricular tachycardia, and haloperidol is more mature and stable; research related to arrhythmia, cardiogenic, pharmacoepidemiology, atrial tachycardia, atrial fibrillation, quetiapine, olanzapine, and risperidone may be well developed and become the next research themes; and clozapine, schizophrenia, and antipsychotics have important roles in this research area and are of high research value (Supplementary Figure 2).

## Discussion

### General Information

The first article studying sudden cardiac death from antipsychotic drugs appeared in 1956 and may have been the origin of this subject. The article was authored by S. J. Weinberg and T. J. Haley, and focused on the effects of chlorpromazine on intracerebroventricular injections of the tryptamine hormone thiophene and thiophene-induced cardiac arrhythmias. From 1956 to the present, with some gaps in the intervening years, there has been an overall slow increase in the literature. There has been an upward trend, suggesting that there is significant research space and value in this area of study. From 1999 to 2000, there was a surge in the literature. According to researcher Glassman, during these years a new atypical antipsychotic, sertindole, emerged which was not registered for marketing. However, because it was associated with several cases of sudden death, it drew the attention of research scholars to the historical problem of prolonged QT intervals from antipsychotics, leading to a new wave of research. Patients with schizophrenia already have a mortality rate for sudden death that is nearly three times higher than normal. Antipsychotics may also increase the risk because some antipsychotics may cause prolonged QT time, leading to severe ventricular arrhythmias and predispositions to sudden death and autonomic dysfunction (51). Compared with no antipsychotics, long-term antipsychotic use treatment has a lower mortality rate (33). Thus, it can be concluded that the high mortality rate in patients with schizophrenia may be partly caused by the influence of multiple factors such as long-term negative health habits, metabolic disorders associated with the disease and treatment, and the consequent increased frequency of cardiovascular disease.

**FIGURE 6:**
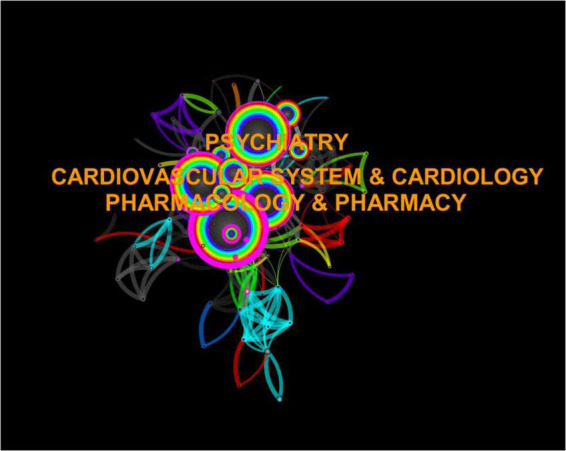
Category network visualization.

**FIGURE 7:**
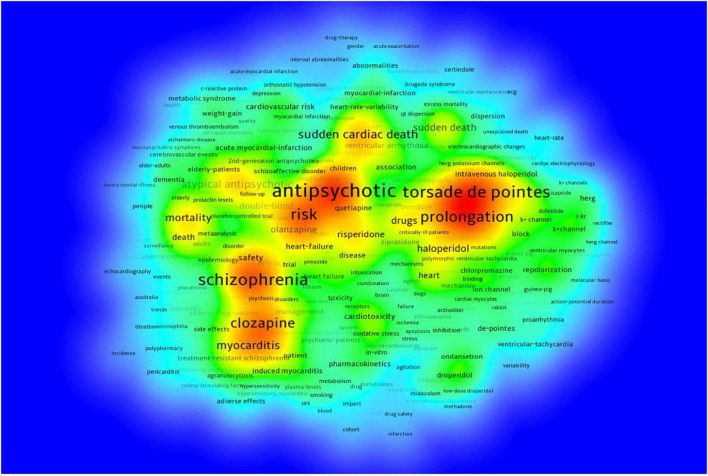
Keyword density visualization.

Previous studies have shown that researchers found that prolongation of QTc from antipsychotic use was associated with sudden death. Subsequent studies meticulously investigated rectifier potassium channels, which paved the way for further studies regarding dependent blockade. Additionally, several studies have confirmed that increased dosage of atypical antipsychotics increases the risk of sudden death. Thus, the doubling of the number of articles from 1999 to 2003 may be related to the above, but predictors are not a substitute for clinical data. Pharmacology and pharmacy, psychiatry, cardiovascular system and cardiology, and cardiac and cardiovascular systems are now the top four disciplines studying the effects associated with sudden cardiac death from antipsychotic drugs. They are closely linked to each other and there is little difference in the number of articles published between the fourth- and third-placed studies (Figures 6, 7).

### Cluster Research

#### Cluster 0

##### Research Base

Clozapine is a very effective drug for treating psychiatric disorders and is popular in clinical practice, but adverse reactions in clinical patients and studies have shown that clozapine use can cause myocarditis and cardiomyopathy (19). However, the evidence that clozapine induces myocarditis or causes cardiomyopathy has not been fully established. Such studies have evaluated the results based on a certain number of subjects with increasing duration of use and increasing dose, and have shown an increased incidence. The first case of myocarditis during clozapine treatment was reported in 1980 and was associated with an overdose (25). In cases of suspected clozapine-induced myocarditis, fever, tachycardia, shortness of breath, and chest pain were the most common symptoms, and C-reactive protein, eosinophil count, and troponin levels were elevated to varying degrees in more than half of the patients. In patients with schizophrenia and bipolar disorder, they also receive concomitant treatment with adjunctive mood stabilizers such as valproic acid and lithium, and evidence supports this as an important risk factor for clozapine myocarditis. Therefore, it is important to be aware of the potential for adverse reactions arising from each other as a result of polypharmacy to avoid being misdiagnosed as a new medical problem and adding an unnecessary risk burden to the patient. Cardiovascular MRI can replace endomyocardial biopsy by virtue of a non-invasive method for timely diagnosis of myocarditis (26, 27, 31). The researchers propose active monitoring for 4 weeks and recommend discontinuing clozapine if troponin exceeds normal or more than twice the upper limit of C-reactive protein levels.

##### Research Front

Clozapine induces myocarditis in the early stages of use and causes cardiomyopathy in the middle and late stages of use, with the former having a risk of death of up to 30%. The incidence of clozapine-induced myocarditis varies widely among studies (52). An autopsy report showed that clozapine-induced myocarditis was associated with rapid titration and that this incidence could be reduced by using a slow titration (29). In contrast, another study showed that the lack of cardiac testing was the main reason for the conflicting results. In cases of suspected clozapine-induced myocarditis, the annual initiation rate of cardiac monitoring increased and so did the incidence of clozapine-induced myocarditis (32). One study concluded differently in 2018 versus 2021 that adverse cardiac reactions in Danish outpatients starting clozapine therapy are extremely rare, and these rates appear to be comparable to those observed with other antipsychotics (53). More cases of clozapine-associated myocarditis were reported in men than in women, at a ratio of 6:1 (54). This seems to weaken the risk studies on clozapine use even more, suggesting that researchers are paying more attention to factors other than the drug itself, with more emphasis on features such as geographical differences and patient sex factors. In this respect, it also suggests that the benefits of clozapine as an antipsychotic far outweigh its adverse consequences and that its use should be continued in the presence of mild disease. Of course, in cases of critical left ventricular dysfunction, clozapine should be suspended without further consideration. Therefore, effective monitoring protocols for early identification and prevention have an important role in research. Current monitoring protocols include slow titration, close cardiac monitoring, and reduction of polypharmacy (54). In the early risk period of clozapine-induced myocarditis, monitoring results using inflammatory markers, such as C-reactive protein, and cardiac damage indicators troponin and brain natriuretic peptide are inconsistent (28). Therefore, investigators should not focus on the need to discontinue clozapine, but rather to provide more effective recommendations for monitoring. The historical research has been relatively stable. This research area has been very active up to 2022, so it is likely to remain this way as a highly engaging research topic.

#### Cluster 1

##### Research Base

In the 1990s, second-generation antipsychotics were used in the clinical treatment of patients with schizophrenia, and their use has been gradually increasing (55). Simultaneously, the risk of severe ventricular arrhythmias and sudden cardiac death was higher in first-generation antipsychotic users than in non-antipsychotic users. Second-generation antipsychotic users also had a higher rate of sudden cardiac death than non-antipsychotic users (56). Generation 1 and 2 drugs had similar dose-dependent increased risks of sudden cardiac death, with their rates being more than twice that of non-users. This suggests that clozapine remains at the forefront of research in future relevant studies. Compared with other drugs, clozapine has the lowest risk of sudden cardiac death (33). Among the second-generation drugs, four had better overall efficacy than the first-generation drugs: amisulpride, clozapine, olanzapine, and risperidone. Among the commonly used therapeutic agents, quetiapine had the highest overall mortality rate compared with fenethylline, while clozapine had a lower suicide rate and the lowest risk of death. Studies have shown that clozapine is the only antipsychotic associated with the development of myocarditis. In an Australian study, 116 case reports of suspected myocarditis were identified in clozapine-treated patients. Clozapine dosages of 100 to 400 mg were prescribed in more than 90% of cases, and 10.3% of patients died. The development of myocarditis is uncommonly associated with clozapine, while in this case, myocarditis is usually fatal, and sometimes occurs early in relatively young patients after treatment initiation (15, 34, 57). Of course, there are some limitations to statistical studies. This may be confounded by factors associated with the use of antipsychotic drugs, including somatic autoimmune diseases, mood disorders, smoking, and concomitant use of other antiarrhythmic drugs. There are also differences in drug use, consumption, and availability of marketing authorization in different countries. Although many strategies have emerged, each method lacks predictive value and these studies suggest that case reports remain the most important source of evidence.

##### Research Front

In older patients (age ≥50 years) taking psychiatric medications, first-generation antipsychotics were associated with a moderately increased risk of serious adverse cardiac events compared with second-generation medications (39). Cardiac adverse events included thromboembolism, myocardial infarction, cardiac arrest, and ventricular arrhythmias. However, age did not have a significant effect in the study. Antipsychotics also have the potential to cause embryonic cardiac bradycardia (37). In pregnant women, where optimal heart rate states exist at different stages of fetal development, reduced rates should be considered unfavorable and may lead to fetal growth retardation, malformations, or spontaneous abortion. This depends on the severity and duration of hypoxia associated with bradycardia. Although antipsychotic drugs can cause heart blockage, inhibition of sinus node activity cannot be excluded as a cause of bradycardia in studies on embryos from pregnant rats. The experimental subjects were rats and species differences are to be expected. At the end of human pregnancy, transfer of antipsychotics from mother to placenta can be examined by comparing maternal and neonatal plasma levels. The mean placental passage rate was 72% for olanzapine, 66% for haloperidol, and 24% for quetiapine (38, 40).

#### Cluster 7

##### Research Base

Various reports emerged in the 1970s and 1980s that excessive QT interval prolongation and tip-twist ventricular tachycardia were associated with the use of various therapies. This was used not only in cardiac studies, but also in studies of antipsychotics. The prevailing view driving clinical care and pharmacological modulation is that cardiac repolarization represents a balance between inward currents (primarily through calcium and sodium channels) and outward currents (primarily through fast and slowed delayed rectifier potassium channels) with fast delayed rectifier channels. This is the primary mechanism by which drugs prolong individual action potentials that manifest on the electrocardiographic surface as prolonged QT intervals (41). Almost all medications that prolong QT interval also block hERG potassium ion channel (24). Additionally, this association was more significant in patients with short-term use, and the risk of ventricular tachycardia and sudden cardiac death was highest for antipsychotics with potassium channel blocking effects of highly effective hERG. Antipsychotic use is associated with a 1.53-fold increased risk of sudden death (41). Antipsychotics at increased risk included haloperidol, prochlorperazine, thioridazine, olanzapine, quetiapine, risperidone, and sulpiride. Studies suggest that pharmacological safety precautions recommend monitoring the corrected QT (QTc) interval, a measure of the duration of ventricular repolarization. Prolonged QT is associated with the risk of arrhythmias, which can lead to early post-depolarization, cause cusp reversal, and result in ventricular fibrillation and, ultimately, sudden cardiac death (58). Numerous population studies have shown relationships between QTc and all-cause mortality, cardiac mortality, and sudden cardiac death (59, 60). To properly interpret the various consequences that result, QT intervals should be appropriately rate corrected to compare values obtained from measurements at different time points and at different heart rates. The current clinical standard is the most widely used Bazett formula. According to the current clinical criteria, Bazett overestimates the number of patients with a potential risk of QTc prolongation, which may lead to unnecessary safety testing and thus non-use of the preferred drug that should be selected for the patient. The Fridericia and Framingham correction formula showed the best correction rate and significantly improved the prediction of 30-day and 1-year mortality (42–45).

##### Research Front

Researchers performed a random-effects direct frequency meta-analysis of pooled data from randomized controlled trials and assessed the quality of the evidence using a graded assessment of recommendations. The evidence suggests that aripiprazole, brexpiprazole, or olanzapine do not increase the QT interval; ziprasidone increases the QT interval and QT interval prolongation in the setting of drug overdose, while risperidone and quetiapine are associated with increased odds of QT interval prolongation and ventricular tachycardia. To avoid QT prolongation and reduce the risk of ventricular tachycardia, clinicians may recommend licensed doses of aripiprazole or olanzapine, which are needed to establish long-term comparative safety (49). The contribution of genetic variation should also be considered when assessed in light of current evidence. Two genetic variants were detected in a forensic autopsy: in the gene encoding the human αT-linked protein and in the gene encoding the RYR2 protein. Both variants were associated with arrhythmogenic right ventricular dysplasia. The RYR2 variant was also associated with autosomal dominant catecholaminergic polymorphic ventricular tachycardia. Dysfunctional RYR2 protein can induce abnormal spontaneous diastolic Ca^2+^ leakage that contributes to delayed post-depolarization formation, which is thought to trigger fatal arrhythmias. Between 2000 and 2021, 115 reported cases of sudden cardiac death were associated with RYR2 mutations (46–48, 50), with evidence supporting the choice of drugs in future relevant treatments. When assessing the cardiac effects of drugs, licensed doses of aripiprazole or olanzapine are better options in clinical medicine. However, from the forensic anatomical findings, it is important to consider genetic variants and not necessarily draw direct equivalences between drugs and sudden cardiac death. Mutations in the RYR2 gene are a relatively new finding in the study of sudden cardiac death caused by antipsychotics. Perhaps in addition to the pathogenic mechanism of drugs, the sensitivity of the organism itself to drugs may give rise to more in-depth studies. The impact of gene mutations continues to remain fresh and vibrant.

### Keyword Evolution

#### Cluster #0

The most frequent keyword in cluster 0 is “risk,” which is consistent with the theme of this cluster. This keyword had a high level of popularity and good development from 2000 to 2021, reaching its peak around 2010. The keyword flow provides a visual macro-level observation that research on the factors influencing the risk of death with antipsychotic drugs is ongoing, and researchers have been seeking a rational explanation for the association of psychotropic drug side effects with cardiotoxicity and sudden death. The differences between specific antipsychotic drugs are greater than those between first- and second-generation antipsychotics. All antipsychotics contribute to prolongation of the QT interval, which can lead to tip-twisting ventricular tachycardia and sudden cardiac death. This effect is most pronounced with the low potency drugs thioridazine and ziprasidone in a dose-dependent manner, with the incidence of sudden cardiac death in patients taking this antipsychotic being approximately twice that of the general population (4). Phenothiazine thioridazine overdose being more cardiotoxic was concluded as early as 1995 (61). The inhibitory effect of thioridazine on the ether-related gene (hERG) channel, which causes voltage-dependent changes, is a mechanism that likely contributes to arrhythmias and sudden death (62). A study of total mortality from antipsychotic drugs in an experiment with 29,823 schizophrenic patients suggested that schizophrenic patients using antipsychotic drugs had mortality rates more than 40% lower than those without antipsychotics. Long-acting injectable second-generation antipsychotics and oral aripiprazole had the lowest mortality rates (63). Although this trial did not clearly and directly show that the effect of drug use on mortality factors was cardiac in origin, this study indirectly suggests that the route of administration is relevant regardless of whether the mortality factor is cardiac in origin. Thus, the type of drug, intrinsic activity, dose, and route of administration are important risk factors for adverse cardiac effects and even sudden death.

#### Cluster #1

The most frequent keyword in cluster 1 is “prolongation.” The evolution of keywords in the timeline, such as cell, k + channel, IKr, and rectifier, can be observed in this cluster based on the microscopic perspective of cellular molecular mechanisms. An early keyword on the timeline, hERG refers to the gene KCNH2, which encodes a potassium channel whose most common role is its potential activity on the heart by coordinating the heartbeat (64). When the channel’s ability to mediate electrical currents through the cell membrane is inhibited, it can lead to the potentially fatal disease known as prolonged QT interval syndrome (65, 66). Prolonged QT interval is often considered to be a marker of cardiac arrhythmia (67). Many antipsychotic drugs can prolong the QT interval and are associated with tip-twisting ventricular tachycardia. This may provide a basis for studying the mechanism by which antipsychotics cause sudden cardiac death. The opposite view has also been proposed, suggesting that hERG measurement alone does not adequately predict the risk of arrhythmogenicity (68). Using repolarization or ion current assays, a concentration-dependent “signal” is often considered evidence of arrhythmogenic risk (69). Especially at higher drug concentrations, multiple drug effects can mask or modulate the potentially deleterious effects of hERG current inhibition. Studies have shown that it is difficult to assess potential arrhythmia risk based on concentration-dependent drug effects in two common *in vitro* preclinical assays. These findings underscore the utility of using natural tissues as the target of drug effects on multiple cardiac ion channels along with integrally measured repolarization studies (68). Chronic drug exposure to phosphatidylinositol 3-kinase (PI3K) inhibitors used in cancer can prolong the QT interval by inhibiting potassium currents and increasing late sodium currents in cardiac myocytes. Some, but not all, drugs designated as arrhythmogenic IKr blockers can trigger arrhythmias by increasing late sodium currents via the PI3K pathway. These data identify potential mechanisms of individual susceptibility to proarrhythmias and highlight the need for a new way to screen for QT-prolonging and arrhythmogenic drugs. This study seems to suggest that the conclusion that antipsychotics cause arrhythmias by inhibiting potassium channel currents or prolonging the QT interval is validated by the need for multifaceted support, that concentration dependence is not established as an indicator of arrhythmia *ipso facto*, and that effects of the PI3K pathway can also produce arrhythmias (70–72). Thus, it does not seem possible to confirm that only antipsychotics themselves can cause arrhythmias through a single effect of hERG that leads to the final sudden death outcome.

#### Cluster #5

The earliest keyword node in the cluster 5 timeline was “ventricular tachycardia” and the most frequent keyword node was “antipsychotic drug.” Ventricular tachycardia is a prominent risk side effect of antipsychotic drugs. In contrast, tip-twisting ventricular tachycardia is an uncommon but fatal arrhythmia. The key literature with the highest number of citations under both nodes is Drew et al. (73). Torsade de Pointes (TdP) is a term coined by researcher Dessertenne (74). However, in some cases, TdP degenerates into ventricular fibrillation and leads to sudden cardiac death. In patients with drug-induced QT interval prolongation syndrome, the QT interval may be normally sinus rhythm prolonged during and without adverse effects, but after suspension, the QT interval prolongation and T-U aberrations become significantly larger and trigger TdP (73). While antipsychotics such as chlorpromazine, haloperidol, thioridazine, and olanzapine can trigger TdP, a prolonged QT interval does not necessarily equate to arrhythmia (75). At the same time, many non-antiarrhythmic drugs are also associated with TdP. For some drugs, including methadone, thioridazine, and haloperidol, multiple case reports have confirmed arrhythmia. Although it is difficult to determine the absolute incidence of TdP from reports of these non-antiarrhythmic drugs, the incidence is generally considered to be lower than that reported for antiarrhythmic drugs (73). For individuals at low risk for arrhythmias, ECG may not be required. Studies have shown that individuals with a risk score of 2 or more should have an ECG before starting a drug that may prolong QTc or starting a drug with a lower risk. Antipsychotics are not equal in causing QTc prolongation. The node with a higher frequency of formation near the middle of the year is “sudden cardiac death,” and the risk of death was determined at 30, 60, 120, and 180 days after the initial dosing of antipsychotics in patients with dementia (76). In summary, recommendations can be made for clinical antipsychotic practice: ECG is a means of observing QT interval prolongation in patients, and the use of antipsychotics is inherently risky. For patients who are already at risk for arrhythmias, risk assessment and ECG should be made before deciding on the use of antipsychotics as a means of preventing further QTc prolongation after drug use. Reducing the risk of TdP can also help avoid a higher risk of sudden cardiac death for the patient. Recent study also showed that wrist-worn smart watches can also be used to monitor the patient’s electrocardiogram (ECG) to detect potential atrial arrhythmias (77).

### The R Programming Language-Thematic Map

The research trends illustrated by the comprehensive analysis, i.e., the cluster analysis, keywords evolution analysis were in approximate agreement with those illustrated in the thematic map. In particular, words located in the fourth quadrant of the thematic map possessed the potential for development in this field, including haloperidol, olanzapine, clozapine, QT interval. Those words were corroborated by the cluster 0 in cluster analysis, cluster #3, cluster #4, and cluster #5 in keyword evolution analysis. Therefore, related studies may continue to play an important role in this field and are of high research value.

## Conclusion

In conclusion, in studies on the cardiac effects associated with antipsychotics on sudden cardiac death, there are more clinical trial studies in Sweden and Denmark, whereas China lacks certain data support. In this article, we analyze the research literature between 1956 and 2022 using calculations and visualizations from CiteSpace and VOSviewer. We also describe in detail the evolutionary trajectory of the knowledge structure in this field over the years from macro to micro from citations, further highlighting possible future research trends. Although medication use increases the risk of sudden cardiac death to some extent, the overall mortality rate without the use of antipsychotic medication is higher than with the use of antipsychotic drugs. The adherence to medication for schizophrenia is only of high clinical significance because of various external and internal factors. By analyzing the articles, we believe that the risk of clozapine use, mainly its cardiotoxicity, is a hot topic of current research. The diagnosis and treatment of the cardiac effects of antipsychotics is of great importance, and involves assessing the risks and benefits of drug selection in psychiatric patients. This necessitates the discarding of subsequent therapeutic drugs and is of high clinical utility. Additionally, this has important implications for forensic research. The results of our bibliometric analysis are more objective and unbiased than traditional review articles written by experts. However, this study has its limitations, although it has better objectivity from these data. To meet the requirements of data analysis, we only included relevant data from the WOS database. Therefore, more databases should be covered in future studies to increase the rigor of the study.

## Data Availability Statement

The raw data supporting the conclusions of this article is available from the public database (Web of Science).

## Author Contributions

TY conceived and designed the study. MW and YM analyzed the data and wrote the initial draft of the manuscript. ZS, LJ, XZ, XW, and ZH collected the data. HL contributed to refining the ideas. All authors involved in revising the manuscript.

## Conflict of Interest

The authors declare that the research was conducted in the absence of any commercial or financial relationships that could be construed as a potential conflict of interest.

## Publisher’s Note

All claims expressed in this article are solely those of the authors and do not necessarily represent those of their affiliated organizations, or those of the publisher, the editors and the reviewers. Any product that may be evaluated in this article, or claim that may be made by its manufacturer, is not guaranteed or endorsed by the publisher.

## Supplementary Material

Supplementary Figure 1 **(A)** Cluster #0 of keyword timeline view- “risk.” **(B)** Cluster #1 of keyword timeline view- “hERG.” **(C)** Cluster #5 of keyword timeline view- “drug-induced QT prolongation.”

Supplementary Figure 2 The R programming language-thematic map by Author keywords.
