# Supplementary figures and images for: Mapping the Knowledge of Antipsychotics-Induced Sudden Cardiac Death: A Scientometric Analysis in CiteSpace and VOSviewer
Source: Front Psychiatry. 2022 Jul 7;13:925583. doi: 10.3389/fpsyt.2022.925583 (PMC9300900; doi:10.3389/fpsyt.2022.925583)

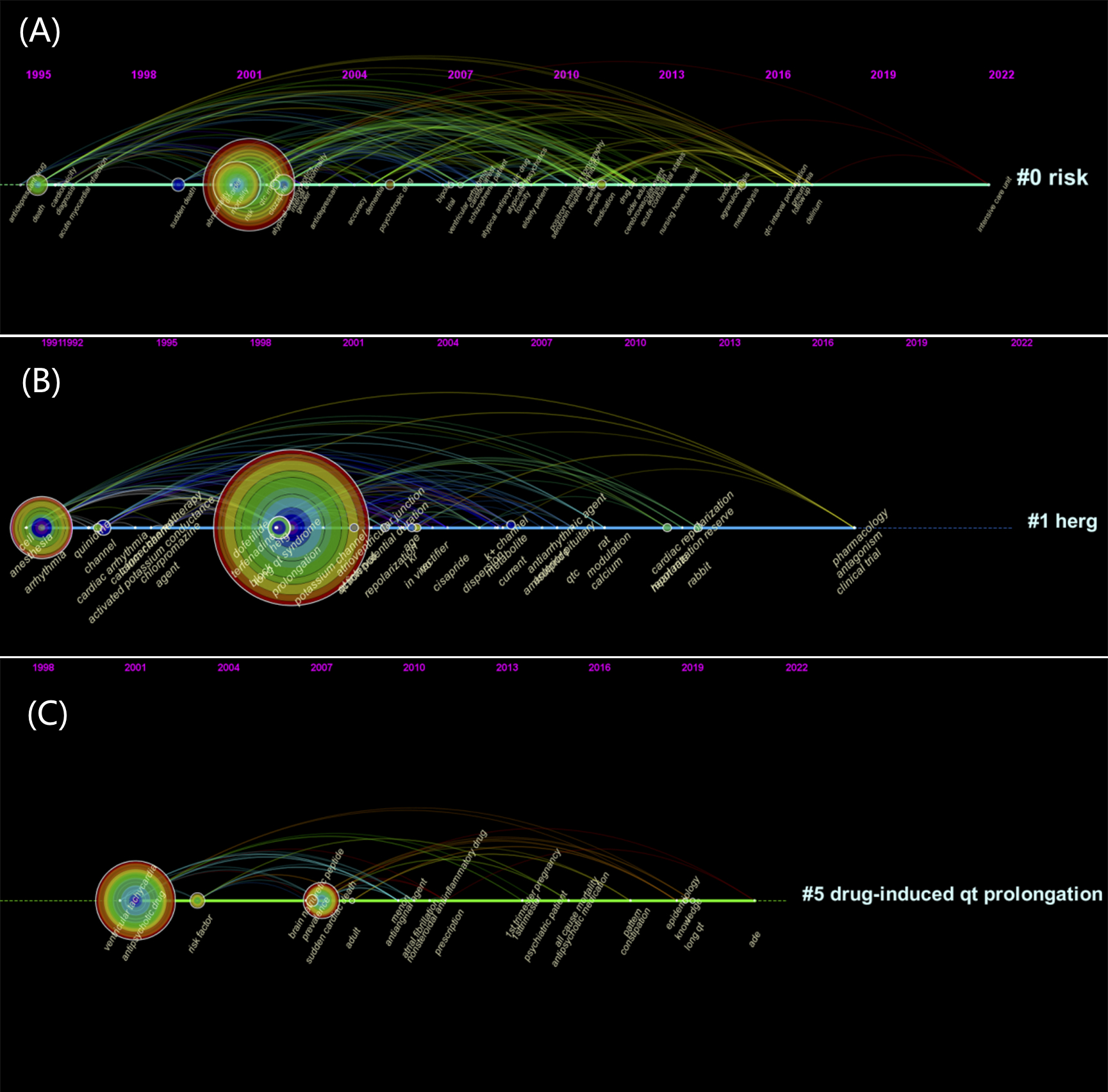

Supplement: Supplementary Figure 1 — (A) Cluster #0 of keyword timeline view- “risk.” (B) Cluster #1 of keyword timeline view- “hERG.” (C) Cluster #5 of keyword timeline view- “drug-induced QT prolongation.” [file Image_1.TIFF]

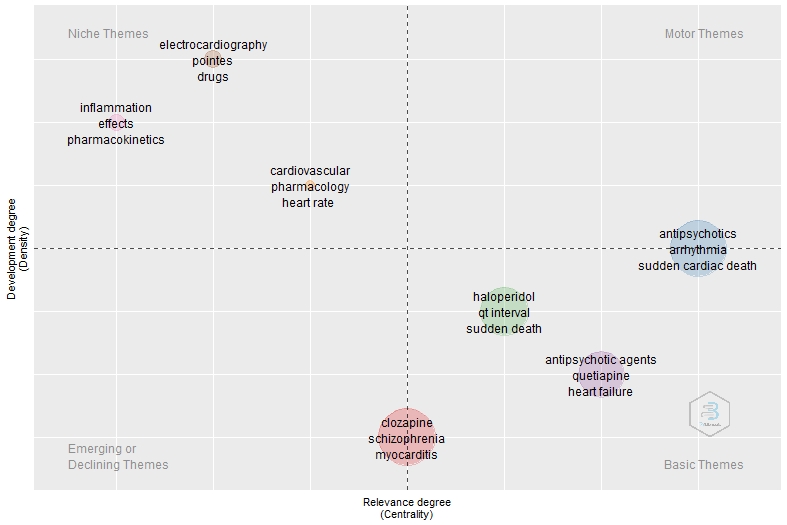

Supplement: Supplementary Figure 2 — The R programming language-thematic map by Author keywords. [file Image_2.JPEG]
